# Supplementary material for: Effects of Olfactory Mucosa Stem/Stromal Cell and Olfactory Ensheating Cells Secretome on Peripheral Nerve Regeneration
Source: Biomolecules. 2022 Jun 11;12(6):818. doi: 10.3390/biom12060818 (PMC9220795; doi:10.3390/biom12060818)
Supplement: Supplementary file 1 [file biomolecules-12-00818-s001.zip › biomolecules-1724491-supplementary.pdf]

Suplementary Material

Table S1: Mean normalized concentration of each biomarker in the CM of OM-MSCs (mean ± SD). *nd* = non-defined.

Biomarker		Mean normalized concentration			
		P4 24h	P4 48h	P7 24h	P7 48h
EGF	Mean	nd	nd	nd	nd
	SD	nd	nd	nd	nd
Eotaxin	Mean	nd	nd	nd	nd
	SD	nd	nd	nd	nd
Fractalkine	Mean	nd	nd	0.040	0.040
	SD	nd	nd	0.00	0.00
G-CSF	Mean	nd	nd	nd	nd
	SD	nd	nd	nd	nd
GM-CSF	Mean	nd	nd	nd	nd
	SD	nd	nd	nd	nd
GRO/KC	Mean	nd	nd	0.08	0.19
	SD	nd	nd	0.00	0.00
IFN-γ	Mean	0.03	0.04	0.06	0.115
	SD	0.01	0.00	0.014	0.06
IL-1α	Mean	nd	nd	nd	nd
	SD	nd	nd	nd	nd
IL-1β	Mean	nd	0.46	0.67	0.84
	SD	nd	0.01	0.23	0.23

Biomarker		Mean normalized concentration			
		P4 24h	P4 48h	P7 24h	P7 48h
IL-2	Mean	nd	nd	nd	nd
	SD	nd	nd	nd	nd
IL-4	Mean	nd	nd	nd	nd
	SD	nd	nd	nd	nd
IL-5	Mean	nd	nd	nd	nd
	SD	nd	nd	nd	nd
IL-6	Mean	nd	nd	nd	nd
	SD	nd	nd	nd	nd
IL-10	Mean	nd	0.01	0.04	0.02
	SD	nd	0.01	0.01	0,00
IL-12p70	Mean	nd	nd	nd	nd
	SD	nd	nd	nd	nd
IL-13	Mean	nd	nd	nd	nd
	SD	nd	nd	nd	nd
IL-17A	Mean	nd	nd	nd	nd
	SD	nd	nd	nd	nd
IL-18	Mean	nd	nd	nd	nd
	SD	nd	nd	nd	nd

Biomarker		Mean normalized concentration			
		P4 24h	P4 48h	P7 24h	P7 48h
IP-10	Mean	nd	0.12	0.28	0.43
	SD	nd	0.01	0.00	0.04
Leptin	Mean	nd	nd	nd	nd
	SD	nd	nd	nd	nd
LIX	Mean	nd	nd	0.02	0.06
	SD	nd	nd	0.00	0.00
MCP-1	Mean	nd	nd	0.05	0.11
	SD	nd	nd	0.00	0.00
MIP-1α	Mean	nd	nd	nd	nd
	SD	nd	nd	nd	nd
MIP-2	Mean	nd	nd	nd	nd
	SD	nd	nd	nd	nd
RANTES	Mean	nd	0.01	nd	nd
	SD	nd	0.00	nd	nd
TNF-α	Mean	nd	nd	nd	nd
	SD	nd	nd	nd	nd
VEGF	Mean	0.01	0.12	0.14	0.48
	SD	0.014	0.02	0.03	0.09

Table S2: Mean normalized concentration of each biomarker in the CM of OECs (mean ± SD). *nd* = non defined.

Biomarker		Mean normalized concentration			
		P4 24h	P4 48h	P7 24h	P7 48h
EGF	Mean	nd	nd	1.00	1.00
	SD	nd	nd	0.05	0.00
Eotaxin	Mean	nd	nd	nd	nd
	SD	nd	nd	nd	nd
Fractalkine	Mean	0.01	0.12	0.30	0.38
	SD	0.01	0.00	0.01	0.02
G-CSF	Mean	nd	nd	0.08	0.92
	SD	nd	nd	0,00	0.11
GM-CSF	Mean	nd	nd	nd	nd
	SD	nd	nd	nd	nd
GRO/KC	Mean	0.03	0.24	0.27	0.92
	SD	0.05	0.05	0.05	0.11
IFN-γ	Mean	nd	0.10	0.46	0.67
	SD	nd	0.15	0.36	0.47
IL-1α	Mean	Mean	0.03	nd	0.96
	SD	SD	0.04	nd	0.06
IL-1β	Mean	0.13	0.21	0.72	0.99
	SD	0.12	0.00	0.30	0.01

Biomarker		Mean normalized concentration			
		P4 24h	P4 48h	P7 24h	P7 48h
IL-2	Mean	nd	nd	nd	nd
	SD	nd	nd	nd	nd
IL-4	Mean	nd	nd	nd	nd
	SD	nd	nd	nd	nd
IL-5	Mean	0.39	0.68	0.95	0.98
	SD	0.01	0.04	0.07	0.04
IL-6	Mean	nd	nd	nd	nd
	SD	nd	nd	nd	nd
IL-10	Mean	nd	0.15	0.08	1.00
	SD	nd	0.17	0.01	0.00
IL-12p70	Mean	nd	nd	nd	nd
	SD	nd	nd	nd	nd
IL-13	Mean	nd	nd	nd	nd
	SD	nd	nd	nd	nd
IL-17A	Mean	nd	nd	0.04	1.00
	SD	nd	nd	0.01	0.00
IL-18	Mean	nd	nd	0.40	0.95
	SD	nd	nd	0.15	0.07

Biomarker		Mean normalized concentration			
		P4 24h	P4 48h	P7 24h	P7 48h
IP-10	Mean	0.03	0.14	0.27	0.78
	SD	0.04	0.04	0.09	0.20
Leptin	Mean	nd	nd	0.49	1.00
	SD	nd	nd	0.01	0.00
LIX	Mean	0.04	0.45	0.61	0.99
	SD	0.04	0.01	0.21	0.01
MCP-1	Mean	0.002	0.02	0.02	0.05
	SD	0.003	3,000	0.002	0.004
MIP-1α	Mean	nd	nd	0.05	0.98
	SD	nd	nd	0.00	0.00
MIP-2	Mean	nd	0.06	nd	1.00
	SD	nd	0.0002	nd	0.004
RANTES	Mean	0.001	0.03	0.14	0.97
	SD	0.002	0.002	0.003	0.04
TNF-α	Mean	nd	nd	nd	nd
	SD	nd	nd	nd	nd
VEGF	Mean	0.006	0.30	0.51	0.95
	SD	0.009	0.05	0.09	0.01

Table S3: Statistical differences in the concentration of biomarkers identified in the CM of OM-MSCs. **a)** Frutalkine; **b)** GRO/KC; **c)** IFN-γ; **d)** IL-1β; **e)** IL-10; **f)** IP-10; **g)** LIX; **h)** MCP-1; **i)** Rantes; **j)** VEGF.

a) **FRUTALKINE**

	P4 24h	P4 48h	P7 24h	P7 48h
**P4 24h**		ns	*	*
**P4 48h**			ns	ns
**P7 24h**				ns
**P7 48h**				

b) **GRO/KC**

	P4 24h	P4 48h	P7 24h	P7 48h
**P4 24h**		ns	ns	ns
**P4 48h**			ns	ns
**P7 24h**				ns
**P7 48h**				

c) **IFN-γ**

	P4 24h	P4 48h	P7 24h	P7 48h
**P4 24h**		ns	ns	ns
**P4 48h**			ns	ns
**P7 24h**				ns
**P7 48h**				

d) **IL-1β**

	P4 24h	P4 48h	P7 24h	P7 48h
**P4 24h**		ns	*	*
**P4 48h**			ns	ns
**P7 24h**				ns
**P7 48h**				

e) **IL-10**

	P4 24h	P4 48h	P7 24h	P7 48h
**P4 24h**		ns	*	**
**P4 48h**			ns	**
**P7 24h**				ns
**P7 48h**				

f) **IP-10**

	P4 24h	P4 48h	P7 24h	P7 48h
P4 24h		*	***	***
P4 48h			**	***
P7 24h				**
P7 48h				

g) **LIX**

	P4 24h	P4 48h	P7 24h	P7 48h
P4 24h		ns	ns	ns
P4 48h			ns	ns
P7 24h				ns
P7 48h				

h) **MCP-1**

	P4 24h	P4 48h	P7 24h	P7 48h
P4 24h		*	***	****
P4 48h			***	****
P7 24h				***
P7 48h				

i) **RANTES**

	P4 24h	P4 48h	P7 24h	P7 48h
P4 24h		ns	ns	ns
P4 48h			ns	ns
P7 24h				ns
P7 48h				

h) **VEGF**

	P4 24h	P4 48h	P7 24h	P7 48h
P4 24h		ns	ns	**
P4 48h			ns	**
P7 24h				**
P7 48h				

Table S4: Statistical differences in the concentration of biomarkers identified in the CM of OECs. **a)** EGF; **b)** Frutalkine; **c)** G-CSF; **d)** GRO/KC; **e)** IFN-γ; **f)** IL-1α; **g)** IL-1β; **h)** IL-5; **i)** IL-10; **j)** IL-17A; **k)** IL-18; **l)** IP-10; **m)** Leptin; **n)** LIX; **o)** MCP-1; **p)** MCP-1α: **q)** MIP-2; **r)** Rantes; **s)** VEGF.

a) **EGF**

	P4 24h	P4 48h	P7 24h	P7 48h
P4 24h		ns	****	****
P4 48h			****	****
P7 24h				ns
P7 48h				

b) **FRUTALKINE**

	P4 24h	P4 48h	P7 24h	P7 48h
P4 24h		**	***	****
P4 48h			**	***
P7 24h				*
P7 48h				

c) **G-CSF**

	P4 24h	P4 48h	P7 24h	P7 48h
P4 24h		ns	ns	***
P4 48h			ns	***
P7 24h				***
P7 48h				

d) **GRO/KC**

	P4 24h	P4 48h	P7 24h	P7 48h
P4 24h		ns	ns	***
P4 48h			ns	**
P7 24h				**
P7 48h				

e) **IFN-γ**

	P4 24h	P4 48h	P7 24h	P7 48h
P4 24h		ns	ns	ns
P4 48h			ns	ns
P7 24h				ns
P7 48h				

f) **IL-1α**

	P4 24h	P4 48h	P7 24h	P7 48h
P4 24h		ns	ns	***
P4 48h			ns	***
P7 24h				***
P7 48h				

g) **IL-1β**

	P4 24h	P4 48h	P7 24h	P7 48h
P4 24h		ns	ns	*
P4 48h			ns	*
P7 24h				ns
P7 48h				

h) **IL-5**

	P4 24h	P4 48h	P7 24h	P7 48h
P4 24h		ns	*	***
P4 48h			*	**
P7 24h				ns
P7 48h				

i) **IL-10**

	P4 24h	P4 48h	P7 24h	P7 48h
P4 24h		ns	ns	***
P4 48h			ns	**
P7 24h				**
P7 48h				

j) **IL-17A**

	P4 24h	P4 48h	P7 24h	P7 48h
P4 24h		ns	**	****
P4 48h			**	****
P7 24h				****
P7 48h				

k) **IL-18**

	P4 24h	P4 48h	P7 24h	P7 48h
P4 24h		ns	*	**
P4 48h			*	**
P7 24h				**
P7 48h				

l) IP-10

	P4 24h	P4 48h	P7 24h	P7 48h
P4 24h		ns	ns	**
P4 48h			ns	*
P7 24h				*
P7 48h				

m) LEPTIN

	P4 24h	P4 48h	P7 24h	P7 48h
P4 24h		ns	****	****
P4 48h			****	****
P7 24h				*
P7 48h				

n) LIX

	P4 24h	P4 48h	P7 24h	P7 48h
P4 24h		ns	*	**
P4 48h			ns	*
P7 24h				ns
P7 48h				

o) MCP-1

	P4 24h	P4 48h	P7 24h	P7 48h
P4 24h		*	*	***
P4 48h			ns	**
P7 24h				**
P7 48h				

p) MCP-1α

	P4 24h	P4 48h	P7 24h	P7 48h
P4 24h		ns	ns	****
P4 48h			ns	****
P7 24h				****
P7 48h				

q) **MIP-2**

	P4 24h	P4 48h	P7 24h	P7 48h
P4 24h		****	ns	****
P4 48h			****	****
P7 24h				****
P7 48h				

r) **RANTES**

	P4 24h	P4 48h	P7 24h	P7 48h
P4 24h		ns	*	****
P4 48h			*	****
P7 24h				****
P7 48h				

s) **VEGF**

	P4 24h	P4 48h	P7 24h	P7 48h
P4 24h		**	**	****
P4 48h			*	***
P7 24h				**
P7 48h				

Table S5: Values of functional deficit (%) obtained performing the EPT test. These tests were performed preoperatively (T0), 1 and 2 weeks after neurotmesis (T1 and T2) and from there every two weeks until week 20 (T20). Results are presented as mean and SD. (*n* = number of animals per group).

**EPT**		**Time**											
		**T0**	**T1**	**T2**	**T4**	**T6**	**T8**	**T10**	**T12**	**T14**	**T16**	**T18**	**T20**
**Group 1: UC**	Mean	7.20	90.00	90.00	85.60	79.20	76.60	69.20	59.00	52.40	44.00	40.60	39.20
**(*n* = 28)**	SD	0.45	2.12	2.12	4.45	7.53	6.07	8.44	6.28	7.92	5.48	5.73	3.35
**Group 2: EtE**	Mean	6.69	74.9	70.6	68.6	65.44	60.27	59.71	49.55	48.03	45.08	38.98	28.19
**(*n* = 5)**	SD	2.51	8.57	11.40	9.92	9.78	9.64	8.70	12.17	9.37	11.47	9.86	17.05
**Group 3: CMOM**	Mean	6.69	74.9	70.6	68.6	65.44	60.27	59.71	49.55	48.03	45.08	38.98	28.19
**(*n* = 6)**	SD	2.51	8.57	11.4	9.92	9.78	9.64	8.70	12.17	9.37	11.47	9.86	17.05
**Group 4: ECMOM**	Mean	10.29	69.67	67.77	67.9	68.54	67.29	66.31	64.45	62.17	47.23	44.31	38.49
**(*n* = 6)**	SD	4.42	8.98	9.77	10.05	9.29	9.37	20.61	8.57	11.7	14.29	7.36	14.71
**Group 5: CMOEC**	Mean	5.41	69.95	66.17	65.15	61.79	61.61	61.61	63.12	49.6	44.73	42.91	40.97
**(*n* = 5)**	SD	7.18	12.15	8.25	3.96	11.41	11.21	11.2	8.46	9.63	8.65	7.22	7.33
**Group 6: ECMOEC**	Mean	1.45	75.07	73.44	71.63	65.55	63.12	62.97	58.96	51.38	50.74	46.66	42.33
**(*n* = 6)**	SD	5.44	6.65	6.47	6.00	11.43	9.99	6.23	5.31	6.80	8.26	8.58	11.13

Table S6: Statical differences observed in EPT test at 20 weeks. (*ns* = no statistically significant differences).

	**EPT**					
	**UC**	**EtE**	**CMOM**	**ECMOM**	**CMOEC**	**ECMOEC**
**UC**		****	****	****	****	****
**EtE**			**ns**	**ns**	**ns**	**ns**
**CMOM**				**ns**	**ns**	**ns**
**ECMOM**					**ns**	**ns**
**CMOEC**						**ns**
**ECMOEC**						

Table S7: WRL values in seconds (s) obtained performing WRL test. These tests were performed preoperatively (T0), 1 and 2 weeks after neurotmesis (T1 and T2) and from there every two weeks until week 20 (T20). Results are presented as mean and SD. (*n* = number of animals per group).

**WRL**		**Time**											
		**T0**	**T1**	**T2**	**T4**	**T6**	**T8**	**T10**	**T12**	**T14**	**T16**	**T18**	**T20**
**Group 1: UC**	Mean	2.39	2.39	2.39	2.39	2.39	2.39	2.39	2.39	2.39	2.39	2.39	2.39
**(*n* = 28)**	SD	1.17	1.17	1.17	1.17	1.17	1.17	1.17	1.17	1.17	1.17	1.17	1.17
**Group 2: EtE**	Mean	4.28	12.00	12.00	11.85	10.48	10.45	10.22	9.82	9.26	8.78	8.57	7.50
**(*n* = 5)**	SD	0.86	0.00	0.00	0.34	2.86	2.12	2.50	2.02	3.07	2.22	0.98	0.87
**Group 3: CMOM**	Mean	2.25	12.00	12.00	8.44	6.22	5.88	4.89	4.06	3.50	2.61	2.33	2.06
**(*n* = 6)**	SD	0.56	0.00	0.00	2.79	1.67	0.98	3.47	1.44	1.26	0.65	0.49	0.57
**Group 4: ECMOM**	Mean	1.94	12.00	12.00	8.83	7.94	7.59	5.72	3.67	3.89	2.83	2.61	2.39
**(*n* = 6)**	SD	0.86	0.00	0.00	1.52	2.02	1.62	2.47	2.22	1.63	0.41	0.53	0.83
**Group 5: CMOEC**	Mean	1.99	12.00	12.00	10.2	9.13	8.73	8.73	8.67	6.87	4.47	4.27	4.07
**(*n* = 5)**	SD	0.89	0.00	0.00	2.16	2.41	2.96	3.22	3.75	2.50	1.24	0.60	0.60
**Group 6: ECMOEC**	Mean	1.94	12.00	12.00	8.56	8.22	7.61	6.72	4.89	4.11	3.78	3.19	2.61
**(*n* = 6)**	SD	0.49	0.00	0.00	1.72	1.66	2.23	3.49	1.87	2.27	1.24	1.03	1.10

Table S8: Statical differences observed in EPT test at 20 weeks. (*ns* = no statistically significant differences).

WRL						
	**UC**	**EtE**	**CMOM**	**ECMOM**	**CMOEC**	**ECMOEC**
**UC**		****	**ns**	**ns**	**ns**	**ns**
**EtE**			****	****	****	****
**CMOM**				**ns**	**ns**	**ns**
**ECMOM**					**ns**	**ns**
**CMOEC**						**ns**
**ECMOEC**						

Table S9: Results of functional recovery through the SFI. These tests were performed preoperatively (T0), 1 and 2 weeks after neurotmesis (T1 and T2) and from there every two weeks until week 20 (T20). Results are presented as mean and SD. (n = number of animals per group).

**SFI**		Time											
		**T0**	**T1**	**T2**	**T4**	**T6**	**T8**	**T10**	**T12**	**T14**	**T16**	**T18**	**T20**
**Group 1: UC**	Mean	-1.45	-1.45	-1.45	-1.45	-1.45	-1.45	-1.45	-1.45	-1.45	-1.45	-1.45	-1.45
**(*n* = 28)**	SD	11.12	11.12	11.12	11.12	11.12	11.12	11.12	11.12	11.12	11.12	11.12	11.12
**Group 2: EtE**	Mean	0.06	-86.73	-86.73	-81.15	-71.8	-67.1	-61.98	-59.59	-58.39	-54.19	-53.99	-46.56
**(*n* = 5)**	SD	3.91	4.22	4.22	17.11	8.12	6.79	11.39	25.98	3.20	20.18	30.30	14.54
**Group 3: CMOM**	Mean	0.76	-62.41	-48.52	-38.48	-38.04	-40.31	-36.98	-27.94	-24.54	-19.89	-13.9	-6.49
**(*n* = 6)**	SD	6.83	25.33	16.04	23.44	16.74	17.23	4.80	18.13	8.68	4.54	15.31	10.56
**Group 4: ECMOM**	Mean	-6.93	-48.57	-40.42	-33.09	-31.61	-30.43	-29.97	-29.47	-26.05	-23.64	-21.31	-21.24
**(*n* = 6)**	SD	4.92	12.26	17.26	10.88	15.79	17.51	21.52	21.74	10.74	6.54	15.54	5.57
**Group 5: CMOEC**	Mean	4.2	-42.88	-44.45	-35.29	-32.95	-30.96	-24.21	-22.46	-20.71	-21.39	-21.29	-17.17
**(*n* = 5)**	SD	11.23	16.8	9.06	20.84	8.60	5.26	12.15	10.54	11.07	6.58	6.66	41.37
**Group 6: ECMOEC**	Mean	15.02	-49.48	-47.54	-45.35	-41.3	-41.18	-33.15	-32.98	-32.15	-29.48	-29,00	-26.82
**(*n* = 6)**	SD	11.62	12.65	6.21	9.97	12.58	22.71	9.49	9.71	17.19	15.7	13.07	15.17

Table S10: Statical differences observed in SFI test at 20 weeks. (*ns* = no statistically significant differences).

SFI						
	**UC**	**EtE**	**CMOM**	**ECMOM**	**CMOEC**	**ECMOEC**
**UC**		****	**ns**	**ns**	**ns**	**
**EtE**			**	**ns**	**ns**	**ns**
**CMOM**				**ns**	**ns**	**ns**
**ECMOM**					**ns**	**ns**
**CMOEC**						**ns**
**ECMOEC**						

Table S11: Results of functional recovery through the SSI. These tests were performed preoperatively (T0), 1 and 2 weeks after neurotmesis (T1 and T2) and from there every two weeks until week 20 (T20). Results are presented as mean and SD. (n = number of animals per group).

SSI		Time											
		T0	T1	T2	T4	T6	T8	T10	T12	T14	T16	T18	T20
**Group 1: UC**	Mean	1.98	1.98	1.98	1.98	1.98	1.98	1.98	1.98	1.98	1.98	1.98	1.98
**(*n* = 28)**	SD	11.87	11.87	11.87	11.87	11.87	11.87	11.87	11.87	11.87	11.87	11.87	11.87
**Group 2: EtE**	Mean	-5.78	-74.44	-73.92	-70.22	-68.04	-59.97	-52.47	-46.56	-44.21	-43.04	-41.91	-33.72
**(*n* = 5)**	SD	12.91	6.83	6.82	6.44	11.02	2.64	27.43	16.79	15.34	12.31	24.55	15.72
**Group 3: CMOM**	Mean	5.68	-65.49	-52.95	-37.73	-38.15	-35.32	-36.49	-29.53	-23.29	-24.7	-11.17	-7.87
**(*n* = 6)**	SD	3.17	15.14	15.93	24.04	16.57	15.63	9.89	16.09	6.28	7.27	11.12	6.54
**Group 4: ECMOM**	Mean	0.06	-45.64	-35.61	-31.43	-30.75	-29.44	-29.25	-26.45	-26.4	-25.95	-21.89	-17.82
**(*n* = 6)**	SD	5.78	10.35	18.25	10.51	19.64	15.56	19.53	14.75	13.5	10.35	8.72	10.11
**Group 5: CMOEC**	Mean	6.89	-44.24	-44.63	-31.72	-30.63	-19.89	-31.51	-25.3	-22.59	-22.22	-21.62	-10.14
**(*n* = 5)**	SD	6.46	17.57	10.61	24.55	9.24	12.57	10.01	14.3	12.67	8.43	8.38	41.08
**Group 6: ECMOEC**	Mean	0.06	-45.64	-35.61	-31.43	-30.75	-29.44	-29.25	-26.45	-26.4	-25.95	-21.89	-17.82
**(*n* = 6)**	SD	5.78	10.35	18.25	10.51	19.64	15.56	19.53	14.75	13.5	10.35	8.72	10.11

Table S12: Statical differences observed in SSI test at 20 weeks. (ns = no statistically significant differences).

SSI						
	**UC**	**EtE**	**CMOM**	**ECMOM**	**CMOEC**	**ECMOEC**
**UC**		***	ns	ns	ns	**
**EtE**			ns	ns	ns	ns
**CMOM**				ns	ns	ns
**ECMOM**					ns	ns
**CMOEC**						ns
**ECMOEC**						

Table S13: Stereological quantitative assessment. The different parameters considered were evaluated in the regenerated sciatic nerve at week 20 (T20) after neurotmesis. Results are presented as mean and SD (*n* = number of animals per group).

Stereological Quantitative Assessment		Density	Total number	Axon diameter (d)	Fiber diameter (D)	Myelin thickness (M)	M/d	D/d	d/D (g-ratio)	Cross-sectional area (mm^2)
**Group 1: UC**	Mean	10167	10200	5.00	8.28	1.64	0.35	1.71	0.60	1.005
**(*n* = 4)**	SD	490.1	1355	0.39	0.40	0.05	0.03	0.07	0.02	0.1258
**Group 2: EtE**	Mean	30072	17423	2.37	3.77	0.70	0.35	1.70	0.60	0.6100
**(*n* = 5)**	SD	5443	2217	0.13	0.21	0.05	0.02	0.03	0.01	0.2115
**Group 3: CMOM**	Mean	20153	12572	3.01	4.42	0.71	0.24	0.68	0.66	0.6258
**(*n* = 6)**	SD	3609	2324	0.33	0.42	0.05	0.14	0.79	0.01	0.0604
**Group 4: ECMOM**	Mean	21631	11462	2.84	4.4	0.78	0.28	0.64	0.65	0.5339
**(*n* = 4)**	SD	1395	3486	0.29	0.63	0.26	0.9	0.45	0.05	0.1727
**Group 5: CMOEC**	Mean	27884	10851	2.73	3.9	0.58	0.21	0.7	0.68	0.3889
**(*n* = 5)**	SD	429	1962	0.72	0.74	0.09	0.12	0.98	0.06	0.0678
**Group 6: ECMOEC**	Mean	22010	11131	2.95	4.28	0.67	0.23	0.69	0.67	0.5033
**(*n* = 4)**	SD	3517	2573	0.27	0.39	0.12	0.44	0.69	0.04	0.0567

Table S14: Statistical differences in the nerve stereological analysis: **a)** Density of fibers; **b)** Total number of fibers; **c)** Axon diameter; **d)** Fiber diameter; **e)** Myelin thickness; **f)** g-ratio; **g)** Cross sectional area. (*ns* = no statistically significant differences).

a) Density

	UC	EtE	CMOM	ECMOM	CMOEC	ECMOEC
**UC**		****	**	***	****	***
**EtE**			***	**	ns	*
**CMOM**				ns	*	ns
**ECMOM**					ns	ns
**CMOEC**						ns
**ECMOEC**						

b) Total number

	UC	EtE	CMOM	ECMOM	CMOEC	ECMOEC
**UC**		*	ns	ns	ns	ns
**EtE**			ns	ns	***	ns
**CMOM**				ns	ns	ns
**ECMOM**					ns	ns
**CMOEC**						ns
**ECMOEC**						

c) **Axon diameter (d)**

	UC	EtE	CMOM	ECMOM	CMOEC	ECMOEC
UC		****	****	****	****	****
EtE			ns	ns	ns	ns
CMOM				ns	ns	ns
ECMOM					ns	ns
CMOEC						ns
ECMOEC						

d) **Fiber diameter (D)**

	UC	EtE	CMOM	ECMOM	CMOEC	ECMOEC
UC		****	****	****	****	****
EtE			ns	ns	ns	ns
CMOM				ns	ns	ns
ECMOM					ns	ns
CMOEC						ns
ECMOEC						

e) **Myelin thickness (M)**

	UC	EtE	CMOM	ECMOM	CMOEC	ECMOEC
UC		****	****	****	****	****
EtE			ns	ns	ns	ns
CMOM				ns	ns	ns
ECMOM					ns	ns
CMOEC						ns
ECMOEC						

f) **d/D (g-ratio)**

	UC	EtE	CMOM	ECMOM	CMOEC	ECMOEC
UC		ns	ns	ns	**	*
EtE			ns	ns	**	*
CMOM				ns	ns	ns
ECMOM					ns	ns
CMOEC						ns
ECMOEC						

g) **Cross-sectional area (mm^2)**

	UC	EtE	CMOM	ECMOM	CMOEC	ECMOEC
UC		**	**	**	***	***
EtE			ns	ns	ns	ns
CMOM				ns	ns	ns
ECMOM					ns	ns
CMOEC						ns
ECMOEC						

Table S15: Muscle Mass Lost in the cranial tibial muscles of the different therapeutic groups compared to healthy contralateral muscles. Healthy Control and Lesion values are expressed in grams (g).

Lost of Muscle Mass		Healthy Control	Lesion	Ratio	% of loss
**Group CMOM (*n* = 6)**	Mean	1.14	0.80	70.86	29.14
	SD	0.14	0.12	7.06	
**Group 4: ECMOM (*n* = 4)**	Mean	1.15	0.67	60.42	39.58
	SD	0.14	0.35	31.84	
**Group 5: CMOEC (*n* = 5)**	Mean	1.33	0.69	53.70	46.30
	SD	0.22	0.27	14.70	
**Group 6: ECMOEC (*n* = 4)**	Mean	1.24	0.54	42.16	57.84
	SD	0.23	0.25	14.53	

Table S16: Statically significant differences in the evaluation of the Tibial Cranial Muscles: **a**) individual fiber area; **b**) minimum Feret's diameter of the muscle fibers (*ns* = no statistically significant differences).

a) **Fibre Area**

	UC	EtE	CMOM	ECMOM	CMOEC	ECMOEC
**UC**		ns	ns	ns	****	****
**EtE**			****	****	****	****
**CMOM**				ns	****	****
**ECMOM**					****	****
**CMOEC**						**
**ECMOEC**						

b) **Minimum Feret's Diameter**

	UC	EtE	CMOM	ECMOM	CMOEC	ECMOEC
**UC**		****	****	****	****	****
**EtE**			****	****	****	****
**CMOM**				****	ns	ns
**ECMOM**					****	**
**CMOEC**						ns
**ECMOEC**						
